# An exploratory study of maximal heart rate determination in endurance athletes: laboratory testing vs. field based

**DOI:** 10.3389/fspor.2026.1806303

**Published:** 2026-04-20

**Authors:** Ådne Ausland, Bence Kelemen, Stephen Seiler

**Affiliations:** 1 Department of Sport Science and Physical Education, Faculty of Health and Sport Science, University of Agder, Kristiansand, Norway; 2 Hungarian University of Sport Science, School of Doctoral Studies; Budapest, Hungary

**Keywords:** endurance athletes, field-based monitoring, heart rate reserve, maximal heart rate, resting heart rate, self-reported data

## Abstract

**Background:**

Accurate assessment of cardiovascular parameters, including maximal heart rate (HRmax), resting heart rate (RHR), and heart rate reserve (HRR), is important for guiding training prescriptions in endurance athletes. Conventional age-based HRmax prediction formulas, particularly the widely used “220—age” equation, remain common despite substantial individual variation and documented underestimation in trained populations. Self-reported field data—defined as the highest heart rate recorded during maximal training or racing efforts—provide an ecologically valid alternative for examining HRmax in real-world settings.

**Methods:**

A cross-sectional study was conducted with 4,375 endurance athletes across multiple disciplines. Self-reported HRmax, RHR, weekly training load, and training history were collected via standardized web-based surveys administered globally from November 2022 to January 2023. Associations were evaluated using Pearson correlations, and the accuracy of age-based HRmax formulas was assessed via Bland–Altman analyses.

**Results:**

Age was strongly inversely correlated with HRmax (*r* = –0.60, *p* < 0.001) and HRR (*r* = –0.66, *p* < 0.001), but only weakly associated with RHR (*r* = 0.06, *p* < 0.001). Age-based formulas underestimated self-reported HRmax by 5–6 bpm (mean bias = –5.8 bpm for Fox; –4.8 bpm for Tanaka), with wide limits of agreement (Tanaka: –18.5 to +9.1 bpm; Fox: –20.2 to +8.6 bpm), indicating substantial individual variability. RHR was moderately inversely correlated with weekly training hours (*r* = –0.23, *p* < 0.001). No significant sex differences were observed. HRR exhibited high inter-individual variability.

**Conclusions:**

This exploratory analysis of self-reported field data demonstrates that age-based HRmax formulas show systematic underestimation and wide individual error in endurance athletes. These findings support the use of individualized, context-specific HRmax assessment, while highlighting the limitations of relying solely on age-based predictions.

## Background

How accurately do endurance athletes know their maximal heart rate (HRmax), the cornerstone of training intensity regulation? Heart rate remains the most popular form of feedback used by endurance athletes to guide and monitor training. Knowing one's maximal heart rate (HRmax), or peak heart rate (HRpeak) across different movement modalities, is central for achieving precise intensity distributions and effective monitoring of the internal “cost” of different exercise prescriptions. HRmax serves as the reference point for prescribing exercise intensity, monitoring training load, and assessing recovery across endurance disciplines (1–4). Its practical value derives from its non-invasive measurability, integration into wearable technologies, and ability to guide the distribution of aerobic, threshold, and high-intensity training zones to optimize adaptation (5).

However, despite its central importance, many athletes and practitioners still rely on estimated HRmax values derived from regression equations, such as “220 minus age” (6) or “208 minus 0.7 × age” (7). While such formulas are convenient and generally valid at the population level, they can introduce large individual errors among trained endurance athletes, whose cardiovascular adaptations diverge from normative reference data (8). Prediction errors of ±10–15 bpm are common, which may meaningfully alter the intended training intensity distribution at the individual level. For example, a 5–6 bpm underestimation may shift an athlete from the intended threshold zone (80–87% HRmax) into lower-intensity training ranges. Although the physiological consequences of such misclassification are plausible, their direct impact on training outcomes remains insufficiently established. This variability underscores the need for individualized HRmax determination to ensure precise training intensity control and recovery assessment (9).

A crucial methodological consideration is distinguishing between HRpeak, the highest heart rate observed during a maximal oxygen uptake (VO_2_max) test, and true HRmax, which reflects the upper physiological limit elicited under deliberate maximal effort (10, 11). These terms are often used interchangeably in applied settings, despite reflecting different physiological constructs. To mitigate this, the survey explicitly asked participants to report HRmax as “the highest heart rate you have ever recorded during a maximal effort in training or competition.” VO_2_max testing primarily targets oxygen consumption endpoints rather than cardiac frequency limits and often terminates when oxygen uptake plateaus or exhaustion occurs, not when maximal heart rate is reached (12). Ingjer (13) demonstrated that HRpeak derived from VO_2_max tests underestimates true HRmax by approximately 5–6 bpm in endurance athletes. Accordingly, protocols specifically designed to elicit HRmax—incorporating extended warm-up and repeated near-maximal efforts—may provide more accurate estimates of maximal cardiac response (14).

Large cohort studies have established both the statistical validity and the limitations of regression-based models. For example, the HUNT Fitness Study (15) derived the equation HRmax = 211–0.64 × age with a standard error of estimate (SEE) of 10.8 bpm, explaining 36% of variance while showing minimal improvement with additional variables (Δ*R*^2^ = 0.007). Although robust at the population level, such formulas leave substantial uncertainty for individual athletes. Similar findings from Londeree and Moeschberger (16) and Whyte et al. (17) confirm age as the primary determinant but reveal wide error margins when applied to endurance populations. Endurance training induces physiological adaptations, such as increased stroke volume, enhanced vagal tone, and attenuated age-related decline that further decouple HRmax from age-based predictions (18).

Laboratory determination of HRmax during a progressive treadmill test to voluntary exhaustion has traditionally been regarded as the gold standard. However, evidence suggests that HRmax is not a fixed physiological constant, but a context-dependent outcome influenced by testing environment, warm-up duration, fatigue level, and motivational state. Ingjer (13) reported that prolonged warm-ups and repeated maximal efforts yielded higher HRmax values, whereas accumulated fatigue reduced peak values. Some studies report slightly higher HRmax values in laboratory settings compared to field conditions, although findings are not entirely consistent across protocols and populations (3). Thus, HRmax assessment should be viewed as protocol-sensitive rather than strictly trait-like, with measurement context shaping observed outcomes (19, 20).

In the modern digital training ecosystem, this traditional laboratory-centric paradigm is evolving. The widespread use of wearables and digital training diaries provides athletes with extensive longitudinal heart rate data across diverse training and competition contexts. Many endurance athletes now contend that their true HRmax is best identified through repeated field efforts rather than a single laboratory test. Despite this, few studies have systematically compared athlete-derived HRmax values from wearables with age-based predictions or laboratory-elicited measures. Existing validations are often limited by small, sport-specific samples and laboratory settings that lack ecological validity (21, 22). Furthermore, the relationships among HRmax, resting heart rate (RHR), and heart rate reserve (HRR), as well as their modulation by autonomic adaptations, remain underexplored (23, 24). Accordingly, the present study leverages a large-scale dataset of self-reported maximal efforts (*N* = 4,375) to characterize real-world HRmax patterns in endurance athletes within an ecologically valid context.

## Materials and methods

### Study design

This descriptive, cross-sectional study investigated quantitative relationships between heart rate parameters—resting heart rate (RHR), maximal heart rate (HRmax), and heart rate reserve (HRR)—and demographic and training-related characteristics in endurance athletes. The variables of interest included age, sex, weekly training hours, and accumulated years of training experience.

Data was collected through two web-based questionnaires administered via Google Forms (Google LLC, Mountain View, CA, USA) between November 2022 and January 2023. The first questionnaire, comprising six items, captured essential demographic and training data, including age, sex, weekly training volume, and training history. The second, a 24-item survey, gathered detailed information on participants' general background, training behaviors, and health status. Both surveys were distributed globally through social media platforms (Twitter, Instagram, Facebook) and targeted outreach to endurance sports clubs and organizations in Norway and internationally.

The target population consisted of self-identified endurance athletes who reported regular training and at least occasional competition participation. From an initial pool of 4,575 responses, 200 were excluded due to implausible values, inconsistent units, or apparent misinterpretations, yielding a final analytic sample of *N* = 4,375 participants. Endurance sports represented in the participants were, cycling (*n* = 1,836, 42%), running (*n* = 1,194, 27.3%), triathlon (*n* = 645, 14.7%), rowing (*n* = 302, 6.9%), cross-country skiing (*n* = 268, 6.1%), swimming (*n* = 22, 0.5%), kayak/canoe (*n* = 8, 0.2%), and others (*n* = 101, 2.3%, mostly biathlon and trail running/cycling). Participants represented Europe (*n* = 2,218, 50.6%), North America (*n* = 1,837, 42%), Oceania (*n* = 198, 4.5%), Asia (*n* = 52, 1.2%), South Africa (*n* = 38, 0.9%), and Africa (*n* = 32, 0.7%).

### Statistical analysis

All statistical analyses were conducted using IBM SPSS Statistics (Version 25, IBM Corp., Chicago, IL, USA). Continuous variables are reported as means ± standard deviations, and categorical variables as frequencies and percentages. The dataset was screened for missing values and evaluated for normality prior to inferential testing.

Group comparisons were performed using chi-square tests for categorical data, while independent samples *t*-tests and one-way analysis of variance (ANOVA) with least significant difference (LSD) *post hoc* tests were applied to continuous variables where appropriate. Pearson correlation coefficients were calculated to quantify the associations between age, training variables, and HR parameters (RHR, HRmax, HRR). Binary correlation analyses were also employed to examine age-related associations with heart rate measures. To evaluate the validity of the Fox (6) and Tanaka et al. (7) equations for predicting HRmax, Bland–Altman analyses were conducted. A two-tailed *p*-value < 0.05 was considered statistically significant throughout.

As exploratory studies are not performed with a specific hypothesis test in mind, the sample size justification cannot be based on an a-priori power analysis (25). However, we report a sensitivity power analysis to indicate which effect sizes could be detected in exploratory statistical tests, with the minimum detectable effect size for correlations at *r* = 0.05 (80% power, *α* = 0.05).

### Ethical considerations

The study complied with the ethical principles of the Declaration of Helsinki. Ethical approval was granted by the Ethics Committee of the Faculty of Health and Sport Science, University of Agder, Norway, and data collection procedures were approved by the Norwegian Centre for Research Data. Both questionnaires were anonymous, collecting no identifiable information to ensure participant privacy and compliance with data protection regulations.

## Results

### Participant characteristics

Participant characteristics are summarized in Table 1. Of the 4,375 endurance athletes included, 74.9% (*n* = 3,280) self-identified as male, 25.0% (*n* = 1,091) as female, and 0.1% (*n* = 4) as non-binary. Male participants were significantly older [mean difference = 2 years, *t*(4371) = 4.52, *p* < 0.001, Cohen's *d* = 0.16, 95% CI (0.09, 0.24)] and heavier [mean difference = 14 kg, *t*(4371) = 46.1, *p* < 0.001, *d* = 1.42, 95% CI (1.35, 1.49)] than female participants. No statistically significant sex differences were observed in RHR, HRmax, or HRR (all *p* > 0.05).

**Table 1:** Physical and training characteristics by sex.

Variable	Male (*n* = 3,280)	Female (*n* = 1,091)	Non-binary (*n* = 4)	All (*n* = 4,375)
Age (years)	44 ± 11*	42 ± 14	38 ± 8	43 ± 11
Years of training	15 ± 10	14 ± 9	8 ± 5	15 ± 10
Endurance training (hours/week)	9 ± 3	10 ± 3	7 ± 2	10 ± 3
Bodyweight (kg)	76 ± 8*	62 ± 7	84 ± 5	73 ± 9
RHR (bpm)	47 ± 5	50 ± 6	51 ± 7	48 ± 6
HRmax (bpm)	183 ± 10	182 ± 10	189 ± 7	183 ± 10
HRR (bpm)	135 ± 11	130 ± 15	138 ± 12	134 ± 12
HRmax Tanaka (bpm)	177 ± 7	179 ± 9	181 ± 5	178 ± 8
HRmax Fox (bpm)	176 ± 11	178 ± 11	182 ± 7	177 ± 11

Values are mean ± SD. RHR, resting heart rate; HRmax, maximal heart rate; HRR, heart rate reserve; HRMAX Tanaka, predicted HRmax using Tanaka's formula; HRmax Fox, predicted HRmax using Fox's formula.

* Significant difference between male and female groups (*p* < 0.05).

Predicted HRmax values based on the Tanaka (7) and Fox (6) formulas were significantly lower than self-reported HRmax values [Tanaka: mean bias = –4.8 bpm, 95% CI (–5.1, –4.5), *t*(4374) = –41.3, *p* < 0.001; Fox: mean bias = –5.8 bpm, 95% CI (–6.2, –5.4), *t*(4374) = –45.7, *p* < 0.001].

### Correlations between heart rate parameters and age

**Figure 1:**
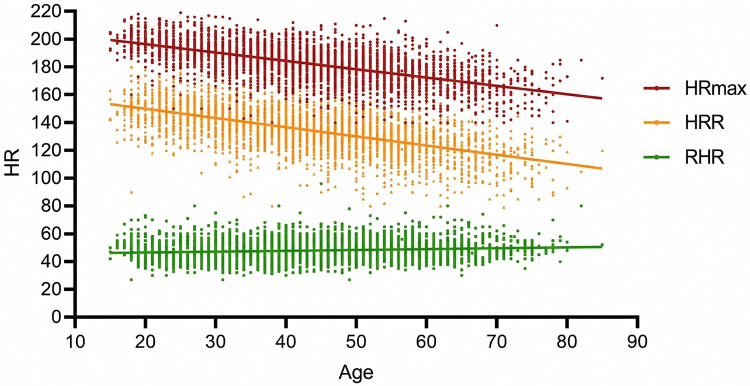
Scatterplots illustrating correlations between age and HRmax (red), HRR (yellow), and RHR (green).

**Figure 2:**
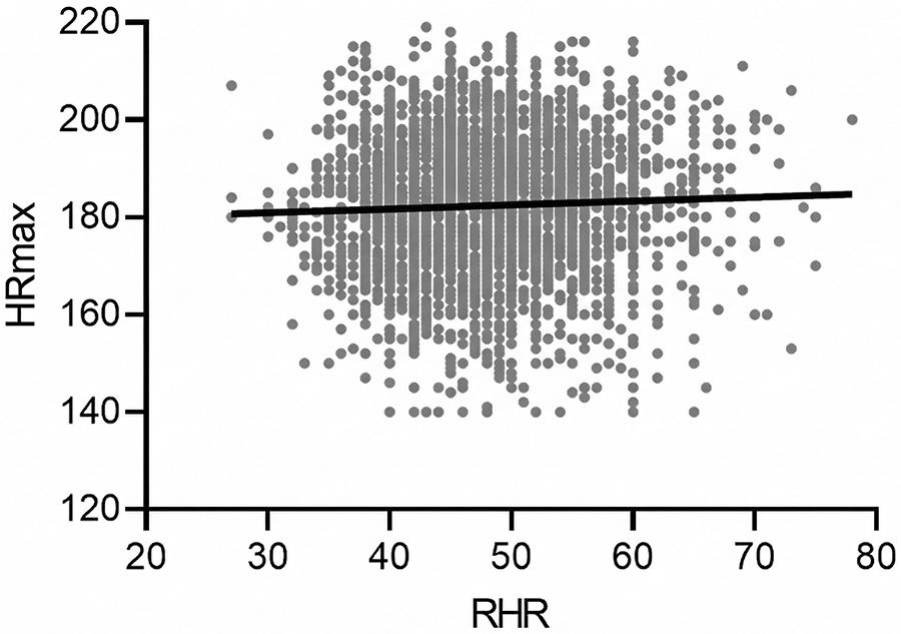
Scatterplot depicting the absence of correlation between HRmax and RHR.

Pearson correlation analyses revealed a strong negative correlation between age and HRmax [*r* = –0.60, *p* < 0.001, 95% CI (–0.62, –0.58)] and between age and HRR [*r* = –0.66, *p* < 0.001, 95% CI (–0.68, –0.64)]. The association between age and RHR was negligible [*r* = 0.06, *p* < 0.001, 95% CI (0.03, 0.09)]. No significant correlation was found between HRmax and RHR (*r* = 0.03, *p* = 0.07). Correlation plots are presented in Figures 1, 2, with full correlation coefficients reported in Table 2.

**Table 2:** Pairwise pearson correlations among heart rate parameters, age, and training characteristics.

Variable	RHR	HRMAX	HRR	Age (y)	Endurance training (y)	Endurance training (h/wk)	Bodyweight (kg)
RHR	1	0.03	–0.45**	0.06**	–0.04	–0.23**	0.03*
HRmax	0.03	1	0.87**	–0.60**	–0.34**	0.11**	–0.09**
HRR	–0.45**	0.87**	1	–0.66**	–0.29**	0.21**	–0.10**
Age (y)	0.06**	–0.60**	–0.66**	1	0.54**	–0.16**	0.16**
Endurance training (y)	–0.04	–0.34**	–0.29**	0.54**	1	0.01	0.03
Endurance training (h/wk)	–0.23**	0.11**	0.21**	–0.16**	0.01	1	–0.16**
Bodyweight (kg)	0.03*	–0.09**	–0.10**	0.16**	0.03	–0.16**	1

Values represent Pearson's *r*. RHR, resting heart rate; HRmax, maximal heart rate; HRR, heart rate reserve; y, years; h/wk, hours per week; kg, kilogram.

* Significant at *p* < 0.05 (two-tailed).

** Significant at *p* < 0.01 (two-tailed). 95% confidence intervals are reported in the text.

### Training characteristics and heart rate measures

Weekly training hours were moderately and inversely correlated with RHR [*r* = –0.23, *p* < 0.001, 95% CI (–0.26, –0.20)]. Training history (years) showed stronger negative correlations with HRmax [*r* = –0.34, *p* < 0.001, 95% CI (–0.37, –0.31)] and HRR [*r* = –0.29, *p* < 0.001, 95% CI (–0.32, –0.26)]. Weak positive correlations were observed between weekly training hours and HRmax (*r* = 0.11, *p* < 0.001) and HRR (*r* = 0.21, *p* < 0.001).

### Validity of age-predicted HRmax formulas

**Figure 3:**
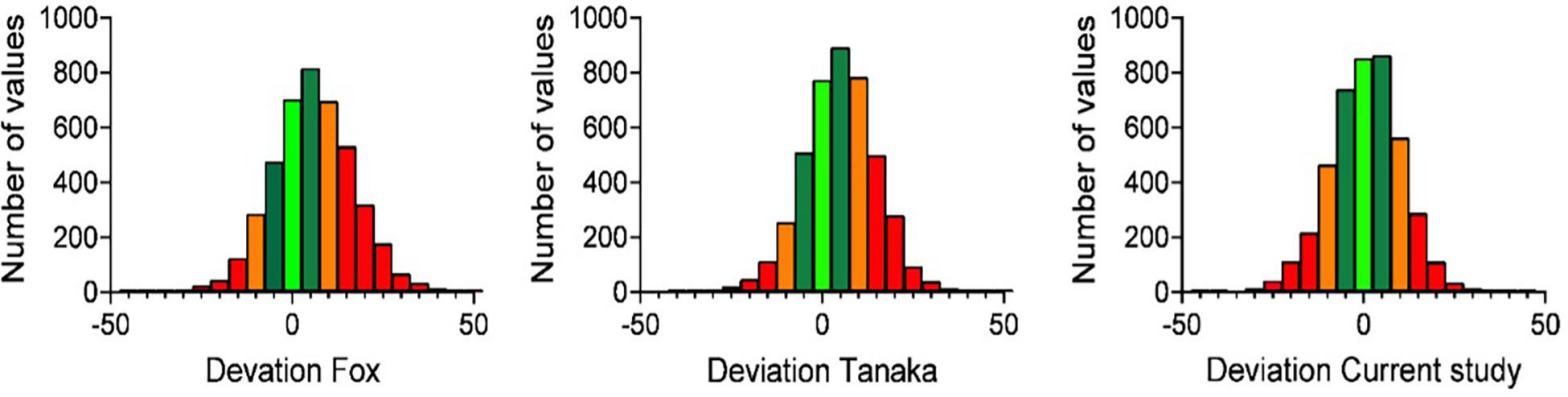
Bland–Altman plots comparing self-reported HRmax with predicted HRmax from Tanaka and Fox equations. Shaded bands indicate ±5 bpm (light green), ±10 bpm (dark green), ±15 bpm (orange), and >±15 bpm (red).

Bland–Altman analyses indicated systematic underestimation of HRmax by both the Tanaka [mean bias = –4.8 bpm, 95% CI (–5.1, –4.5)] and Fox [mean bias = –5.8 bpm, 95% CI (–6.2, –5.4)] equations. Limits of agreement were wide (Tanaka: –18.5 to +9.1 bpm; Fox: –20.2 to +8.6 bpm), reflecting considerable individual variability. Bland–Altman plots (Figure 3) confirmed both over- and underestimation, with discrepancies visually highlighted.

## Discussion

The principal findings of this large-scale exploratory study (*N* = 4,375 endurance athletes) are that age-predicted HRmax formulas systematically underestimate self-reported field values by approximately 5–6 bpm and exhibit wide limits of agreement (±10–15 bpm), confirming substantial individual-level variability in trained populations. Age showed strong inverse correlations with HRmax and HRR, whereas RHR was primarily associated with recent training load rather than chronological age.

These results replicate and extend previous laboratory-based validations [e.g., (15, 26)] to a real-world, self-reported field context. While the observed mean bias is relatively small, the wide limits of agreement and substantial inter-individual variability indicate that age alone is insufficient for precise individual-level training prescription. Recent work by Martin et al. (27) similarly documented small mean biases but wide limits of agreement (∼±18–24 bpm) across varying cardiorespiratory fitness levels, reinforcing that age alone explains only part of HRmax variance. These patterns are consistent with large-scale endurance-specific validations showing systematic underestimation in runners and cyclists (28) and the need for performance-level-specific reference values (29). Endurance training induces physiological adaptations, such as increased stroke volume, enhanced vagal tone, and attenuated age-related decline that further decouple HRmax from age-based predictions (18).

For training prescription, these findings carry direct practical implications: reliance on age-based formulas risks zone misclassification and suboptimal adaptation. Therefore, athletes and coaches should prioritize individualized field-verified HRmax—obtained through repeated maximal efforts in training or competition—over generic equations or single laboratory tests. However, age-based formulas may still serve as a rough initial reference in the absence of individualized data, provided their substantial uncertainty is explicitly acknowledged. Although such misclassification is theoretically plausible, its direct impact on training outcomes remains to be confirmed in longitudinal intervention studies. HRR should be used cautiously as a training anchor given its instability, while RHR offers a sensitive marker of acute load.

Limitations of the study include reliance on self-reported data, which may introduce recall bias or device heterogeneity. Had interviews been possible, clarification of terminology and verification of maximal-effort contexts could have reduced misinterpretations; the present design captures only snapshot self-reports without such validation. The sample, recruited via social media, likely represents a motivated subset of endurance athletes interested in data-driven training rather than the full performance spectrum; however, the large size (*N* = 4,375) and broad disciplinary and geographical representation strengthen generalizability to the global community of self-monitoring endurance athletes. At the same time, the absence of objective performance indicators (e.g., competition level, race results, or VO_2_max) limits precise classification of athlete level, and the sample may include a heterogeneous mix of recreational and competitive individuals.

Statements regarding potential differences between field- and laboratory-elicited HRmax are not derived from the present dataset but are based on prior literature (3, 13). Central to these findings is the protocol-dependent nature of HRmax attainment. VO_2_max test HRpeak does not guarantee true HRmax, as demonstrated by Ingjer (13), who found that a single 3 to 4-minute run yielded HRpeak 5 to 6 beats per minute lower than a protocol with repeated bouts. Ingjer's (13) seminal work established that warm-up duration, the inclusion of repeated near-maximal efforts, and the influence of accumulated fatigue all significantly affect the attainable maximum. VO_2_max protocols, typically designed to assess oxygen transport capacity, tend to underestimate true HRmax by 5–6 bpm because termination criteria are linked to oxygen uptake plateau or volitional exhaustion rather than maximal chronotropic response (10, 30, 31). Prior studies further suggest that field-based efforts may yield slightly lower maximal values than laboratory protocols due to motivational and environmental constraints; however, this pattern was not directly examined in the present dataset (3, 26). This suggests that HRmax reflects not only physiological capacity but also the testing context, a nuance overlooked by static formulas.

For practical application, age-based predictions should serve as a starting point, with HRmax verified through a protocolized approach—e.g., a 10–30-min warm-up followed by 2–3 maximal 3 to 4-min runs, adjusted for individual fitness. Coaches and athletes should report the context of HRmax determination (e.g., lab vs. field, number of efforts) when assigning zones or comparing data across studies, enhancing precision in training prescriptions. In applied monitoring, coaches should exercise caution in using HRR as a training anchor given its instability, while recognizing that RHR can serve as a sensitive indicator of acute training load, albeit subject to modulation by external factors such as sleep, stress, and environmental variation (2, 32, 33). This individualized strategy aligns with emerging evidence favoring field-based monitoring over lab estimates (34).

Additional limitations include the cross-sectional design, which precludes causal inference, and the potential underrepresentation of certain endurance disciplines. For female athletes, the absence of hormonal status data and menstrual cycle phase information restricts the interpretation of sex-specific cardiovascular responses, potentially influencing HRmax and RHR variability due to hormonal fluctuations (35, 36). Strengths lie in the breadth and ecological validity of its sample, encompassing over 4,000 endurance athletes, the simultaneous evaluation of multiple predictive equations, and the rigorous application of Bland–Altman methods to quantify both systematic bias and individual limits of agreement. By leveraging athlete-reported field data, the study captures the realities of endurance training more faithfully than laboratory-based investigations alone.

Future work should critically examine whether the pursuit of standardized laboratory-style protocols is truly warranted for athletes. Our findings, together with prior research, suggest that maximal heart rate is highly context-dependent and influenced by motivation, environment, accumulated fatigue, and testing design (3, 13). Rather than developing increasingly rigid “gold-standard” protocols, greater value may lie in documenting the conditions under which HRmax is elicited and recognizing the inherent variability between field and lab settings. Longitudinal tracking of athletes in their natural training environments, combined with simple contextual information, may provide more ecologically valid insights than one-off standardized tests. Future research should therefore explore the utility of individualized, field-based assessments, complemented by broader autonomic markers such as heart rate variability, to better capture readiness and recovery while moving beyond the assumption that HRmax can or should be fixed to a single standardized number.

## Conclusion

This study showed that age-based HRmax prediction formulas (6, 7) underestimate self-reported field HRmax by ∼5 beats per minute in a large cohort of endurance athletes. Age-based formulas should be abandoned as primary tools; individualized field-based verification is recommended to support training precision. Where feasible, individualized field-based verification of HRmax is recommended to improve training precision, while acknowledging that HRmax itself is context-dependent and influenced by testing conditions. While self-reported data and the cross-sectional design represent methodological limitations, the breadth of the cohort adds ecological validity. The findings question the value of highly standardized laboratory protocols, suggesting instead that HRmax should be viewed as context-dependent and variable. Future directions should emphasize individualized and field-based assessments, transparent reporting of testing conditions, and integration with other markers of athlete readiness. Such approaches may better support training precision and athlete performance than reliance on generic prediction formulas or rigid testing standards.

## Data Availability

The raw data supporting the conclusions of this article will be made available by the authors, without undue reservation.
